# Consumption of Herbal Supplements or Homeopathic Remedies to Prevent COVID-19 and Intention of Vaccination for COVID-19 in Latin America and the Caribbean

**DOI:** 10.3390/tropicalmed7060095

**Published:** 2022-06-08

**Authors:** Guido Bendezu-Quispe, Jerry K. Benites-Meza, Diego Urrunaga-Pastor, Percy Herrera-Añazco, Angela Uyen-Cateriano, Alfonso J. Rodriguez-Morales, Carlos J. Toro-Huamanchumo, Adrian V. Hernandez, Vicente A. Benites-Zapata

**Affiliations:** 1 Escuela de Medicina, Universidad César Vallejo, Trujillo 13001, Peru; gbendezuqu@ucvvirtual.edu.pe; 2 Red Internacional en Salud Colectiva y Salud Intercultural, Mexico City 56690, Mexico; percy.herrera@upsjb.edu.pe; 3 Sociedad Científica de Estudiantes de Medicina de la Universidad Nacional de Trujillo, Trujillo 13011, Peru; jbenitesm@unitru.edu.pe; 4 Grupo Peruano de Investigación Epidemiológica, Unidad para la Generación y Síntesis de Evidencias en Salud, Universidad San Ignacio de Loyola, Lima 15012, Peru; 5 Facultad de Ciencias de la Salud, Universidad Científica del Sur, Lima 15067, Peru; 6 Instituto de Evaluación de Tecnologías en Salud e Investigación—IETSI, EsSalud, Lima 14072, Peru; 7 Escuela de Enfermería, Universidad Privada San Juan Bautista, Lima 15067, Peru; 8 Medecins Sans Frontieres, Health Politics, 1050 Brussels, Belgium; dra.uyen@gmail.com; 9 Latin American Network of Coronavirus Disease 2019 Research (LANCOVID), Pereira 660003, Colombia; alfonso.rodriguez@uam.edu.co; 10 Grupo de Investigación Biomedicina, Faculty of Medicine, Fundación Universitaria Autónoma de las Américas, Pereira 660003, Colombia; 11 Facultad de Ciencias de la Salud, Universidad Peruana de Ciencias Aplicadas, Lima 15023, Peru; ctoro@usil.edu.pe; 12 Health Outcomes, Policy, and Evidence Synthesis (HOPES) Group, School of Pharmacy, University of Connecticut, Storrs, CT 06269, USA; adrian.hernandez-diaz@uconn.edu; 13 Unidad de Revisiones Sistemáticas y Metaanálisis, Guías de Práctica Clínica y Evaluaciones Tecnológicas Sanitarias, Vicerrectorado de Investigación, Universidad San Ignacio de Loyola (USIL), Lima 15024, Peru; 14 Unidad para la Generación y Síntesis de Evidencias en Salud, Universidad San Ignacio de Loyola (USIL), Lima 15012, Peru

**Keywords:** homeopathy, herbal medicine, vaccination, COVID-19, Latin America

## Abstract

Users of complementary and alternative medicine (CAM) have a lower intention to receive vaccines. Furthermore, Latin America and the Caribbean (LAC) region are among the most affected areas by the COVID-19 pandemics and present a high proportion of CAM users. Therefore, this study evaluates the association between the consumption of herbal supplements or homeopathic remedies to prevent COVID-19 and the intention to vaccinate against COVID-19 in the LAC region. We conducted a secondary data analysis of a Massachusetts Institute of Technology (MIT) survey with Facebook to assess COVID-19 beliefs, behaviours, and norms. Crude and adjusted prevalence ratios (aPR) with their respective 95% confidence intervals (95% CI) were calculated using generalized linear models of the Poisson family with the log link function. The prevalence of the use of products to prevent COVID-19 was the following: consumption of herbal supplements (7.2%), use of homeopathic remedies (4.8%), and consumption of garlic, ginger, and lemon (11.8%). An association was found between using herbal supplements (19.0% vs. 12.8%; aPR = 1.44; 95% CI: 1.30–1.58), the use of homeopathic remedies (20.3% vs. 12.3%; aPR = 1.58; 95% CI: 1.25–1.98), and the consumption of garlic, ginger, and lemon (18.9% vs. 11.9%; aPR = 1.55; 95% CI: 1.50–1.61) and non-intention to vaccinate against COVID-19. In the LAC population, there is an association between using herbal supplements, using homeopathic remedies and consuming garlic, ginger, and lemon to prevent infection by COVID-19 and non-intention to vaccinate against this disease. Therefore, it is necessary to design targeted strategies for groups that consume these products as preventive measures against COVID-19 to increase vaccination coverage and expand the information regarding transmission and prevention strategies for SARS-CoV-2.

## 1. Introduction

In the last two decades, three types of coronavirus with a significant impact on global health have emerged in the world, SARS-CoV-2 (COVID-19), SARS (Severe Acute Respiratory Syndrome), and MERS (Middle East respiratory syndrome) [1]. On 11 March 2020, the World Health Organization (WHO) declared the COVID-19 pandemic [2], reporting a total of 251,885,689 cases and 5,079,013 deaths worldwide for this disease up to 11 November 2021 [3].

The therapeutic options to treat patients with COVID-19 are limited, with the development of the vaccine being the most accepted mitigation strategy having more than 200 vaccine candidates to date [4]. Although vaccination is the most cost-effective public health strategy [5,6], for the control of COVID-19, according to some experts, coverage must reach >70% of the population [7]. However, a significant proportion of the world population is reluctant to use the vaccine due to information and misinformation prevalent worldwide regarding vaccines. The reasons for rejection are fear of adverse effects, beliefs, medical mistrust, and structural barriers [8,9,10]. Some studies have estimated that acceptance of the vaccine against COVID-19 during the first year of the pandemic varied from 27.7% to 93.3% in the world [11,12,13]. 

Previous studies show that CAM users have a lower intention to receive vaccines [14,15], including a lower intention to vaccinate their children [16]. During the COVID-19 pandemic, lower vaccination intention has also been described in people and health professionals who use or indicate traditional and complementary medicine [17,18]. However, there is currently insufficient evidence on the efficacy and safety of these therapies in preventing or treating COVID-19 [19].

Complementary and alternative medicine (CAM) is used worldwide, and in countries with limited access to healthcare, it is often the only accessible and available treatment [20]. It is estimated that up to four billion people (representing 80% of the world’s population) living in developing countries use herbal medicinal products as a primary source of medical care [21]. In Africa, up to 80% of the population uses traditional medicine to treat their health problems [22]. However, an estimated 57 countries in the African region face a critical shortage of health workers, with a deficit of 2.4 million doctors and nurses [23]. Africa has 2.3 health workers per 1000 inhabitants, compared to the Americas, with 24.8 health workers per 1000 inhabitants [23]. Likewise, in East Asia, the prevalence of alternative and complementary traditional medicine has been reported in up to 76.7% of the population [24]. However, only 1.3% of the world’s health workforce cares for people who suffer from 25% of the global burden of disease [23]. 

Latin America and the Caribbean (LAC) is one of the regions most affected by the pandemic. Countries such as Mexico, Brazil, and Peru have the highest number of cases and deaths from this disease in the world [3]. It has been described that in LAC, gender, fear of oneself or a family member becoming seriously ill and having depressive symptoms are associated with vaccinating [8]. According to the WHO Regional Office for the Americas (AMRO/PAHO), 71% of the population in Chile and 40% of the population in Colombia use traditional medicine [25], and up to 70% of the population of the entire continent use medicinal plants [26]. Despite the potential benefits that these therapies could represent, their use is associated with a lower intention to vaccinate against COVID-19. This scenario is of utmost importance for LAC, one of the regions hardest hit by the pandemic and the most unequal in the world. Limited access to health services has been compounded by the disruption of services generated by COVID-19 and the appearance of new COVID-19 variants [27]. In addition, the use of CAM is frequent, and to our knowledge, there are no studies on this association. Therefore, the objective of this study was to evaluate the association between the consumption of herbal supplements or homeopathic remedies to prevent COVID-19 and the intention to vaccinate against COVID-19.

## 2. Materials and Methods

### 2.1. Study Design

A secondary analysis was performed using a database compiled by the Massachusetts Institute of Technology (MIT) in collaboration with Facebook. This survey aimed to evaluate beliefs, behaviours, and norms related to COVID-19. Data collection began on 7 July 2020 and ended on 28 March 2021. It was conducted in more than 60 countries and translated into 51 languages. Two versions of the survey were available. First, in countries with a good group of users to sample, a multi-wave survey was conducted continuously for several two-week waves to collect 3000 respondents for each wave. Second, in countries with a limited survey pool, we fielded a snapshot survey with which Facebook aimed to deliver 3000 respondents over two weeks.

### 2.2. Population, Sample, and Sampling

**Figure 1:**
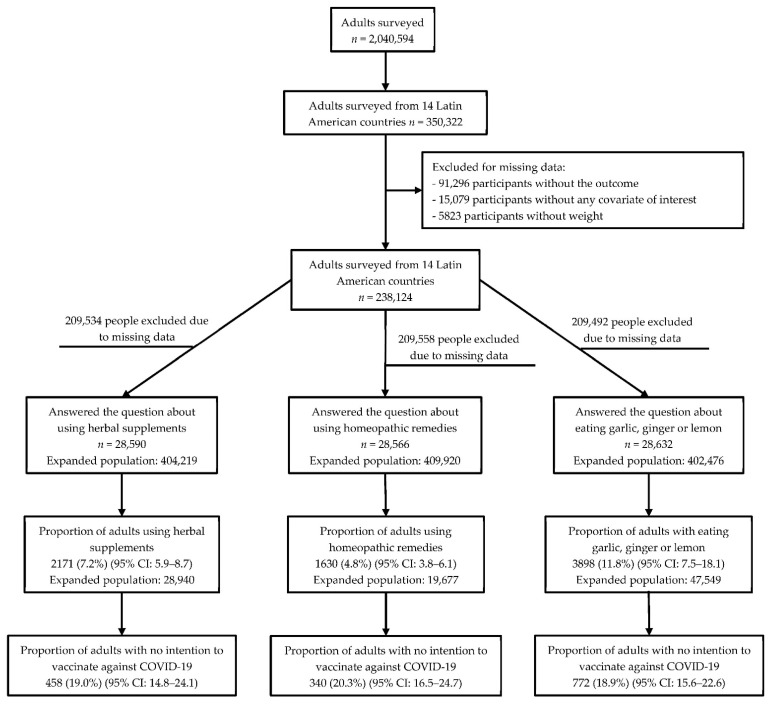
Flowchart of the selection of participants included in the analysis.

The survey included participants aged 18 or older who were Facebook users. In addition, participants who resided in LAC and participated in the survey from 7 July 2020 to 28 March 2021 were included. Furthermore, participants who did not present data for the variables of interest and did not have the weighting factor to perform the corresponding analyses were excluded. Finally, three groups of participants were obtained according to each primary exposure presented (Figure 1).

### 2.3. Variables

The outcome variable was non-intention to be vaccinated, which was operationalized from the question: If a vaccine for COVID-19 becomes available, would you choose to get vaccinated? The possible answers to this question were: yes, no, I do not know, I am already vaccinated. Therefore, the construction of the variable was carried out considering only those who answered yes or no.

There were three exposure variables constructed from the responses to the question: What measures have you taken to prevent infection from COVID-19 in the past week? Sixteen possible response alternatives corresponded to preventive measures against COVID-19 infection and were presented randomly to facilitate the development of the survey. The three exposure variables were constructed considering the responses: (1) the use of herbal supplements, (2) the use of homeopathic remedies, and (3) the consumption of garlic, ginger, and lemon.

Other variables included were gender (male, female, non-binary), age (18–30, 31–40, 41–50, 51–60, 61–70, 71–80, over 80), educational level (less than primary school, primary school, secondary school, college/university, graduate school), area of residence (city, town, village, or rural area) and health status (poor, fair, good, very good, excellent).

### 2.4. Statistical Analysis

The databases were downloaded in text format “.txt” and were imported into the statistical package STATA v15.0 (StataCorp, Texas, USA). Then, all analyses were carried out considering the complex sampling of the survey using the svy command.

A descriptive analysis was performed using absolute frequencies and weighted proportions with their respective 95% confidence intervals (95% CI) according to the survey’s complex sampling. In the bivariate analysis, we used Pearson’s Chi-square test with Rao-Scott correction. To evaluate the association between exposures and outcome (No intention to vaccinate), generalized linear models of the Poisson family with log link function were constructed. The crude prevalence ratio (cPR) and adjusted (aPR) were calculated with their respective 95% CI for the associations studied. The adjustment for confounders was carried out considering an epidemiological approach [8,28]. Collinearity was evaluated using variance inflation factors (VIF), considering a cut-off point of less than 10. Additionally, given the potential selection bias, a comparative analysis was carried out between the participants who had missing or no missing data of each exposure with the other variables of interest, not finding essential differences (Tables S1–S3). A *p*-value less than 0.05 was considered statistically significant for all the statistical tests performed.

### 2.5. Ethical Considerations

All participants provided informed consent at the beginning of the survey. The present study analyzed a secondary database that did not have personal identifiers and respected the integrity of the participants. The data were obtained through access granted by the MIT, Boston, United States of America.

## 3. Results

### 3.1. Selection of the Study Sample

**Figure 2:**
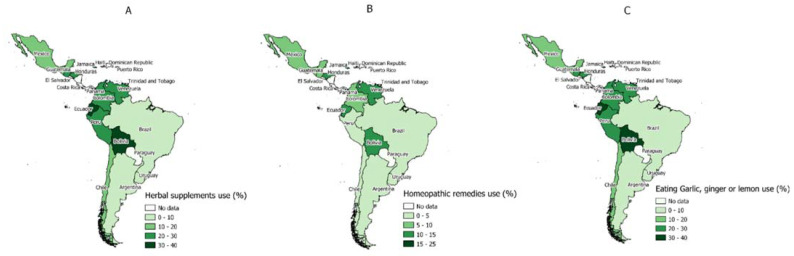
(**A**) Prevalence of use of herbal supplements in adults surveyed from 14 Latin American countries. (**B**) Prevalence of use of homeopathic remedies in adults surveyed from 14 Latin American countries. (**C**) Prevalence of consumption of garlic, ginger, or lemon in adults surveyed from 14 Latin American countries.

The global population surveyed was made up of 2,040,594 Facebook users over 18 years of age. Participants residing in LAC countries (350,322) were included, and all those who did not provide information for our variables of interest were excluded. Finally, the population included presented variations in terms of the type of CAM used: herbal supplements (28,590), use of homeopathic remedies (28,566), and consumption of garlic, ginger, and lemon (28,632) (Figure 2). The participants were then divided into three groups according to the type of exposure.

### 3.2. Characteristics of the Samples Included in the Study

The characteristics of the participants who made up each exposure group were very similar. A higher proportion of male participants, aged between 18 to 30 years, with a high school education level and who lived in the city. Only the group that consumed garlic, ginger, and lemon had a higher proportion of women. The prevalence of *using* herbal supplements was 7.2%, 4.8% for homeopathic remedies, and 11.8% for eating garlic, ginger, and lemon (Figure 1). The non-intention to vaccinate was 13.2% in the group using herbal supplements and 12.7% in both participants using homeopathic remedies and those consuming garlic, ginger, and lemon. In general, all the groups reported a good, very good, or excellent health status (Table 1).

### 3.3. Prevalence of Using Herbal Supplements to Prevent COVID-19 Infection by Each LAC Country

The countries with the highest prevalence in terms of the consumption of herbal supplements were Bolivia (19.6%), Trinidad & Tobago (19.5%), Venezuela (18.8%), Jamaica (17.3%), and Ecuador (15.4%). At the same time, those showing the lowest prevalence of consumption of herbal supplements were Uruguay (2.0%), Argentina (2.6%), Chile (5.6%), Mexico (6.5%), and Brazil (7.9%) (Figure 2A and Table S4).

### 3.4. Prevalence of the Use of Homeopathic Remedies to Prevent COVID-19 Infection by Each LAC Country

Regarding the use of homeopathic remedies, the countries with the highest prevalence were Trinidad & Tobago (21.5%), Bolivia (14.6%), Honduras (12.1%), Jamaica (12.0%), and Venezuela (11.4%). In comparison, the lowest prevalence of these remedies was found in Uruguay (1.8%), Argentina (3.5%), Brazil (4.2%), Peru (4.6%), and Colombia (4.7%) (Figure 2B and Table S5).

### 3.5. Prevalence of Garlic, Ginger, and Lemon Consumption to Prevent COVID-19 Infection by Each LAC Country

Lastly, the prevalence of garlic, ginger, and lemon consumption was more frequent in Ecuador (39.0%), Bolivia (34.1%), Venezuela (29.3%), Honduras (28.9%), and Guatemala (28.8%). On the other hand, the countries with the lowest prevalence of garlic, ginger and lemon consumption were Argentina (7.2%), Brazil (9.0%), Uruguay (9.9%), Chile (11.0%), and Mexico (15.3%) (Figure 2C and Table S6).

### 3.6. Bivariate Analysis According to Each Measure Taken to Prevent COVID-19 Infection

We found a higher proportion of non-intention to vaccinate among herbal supplements users than non-users (19.0% vs. 12.8%; *p* < 0.001), with statistically significant differences according to sex, level of education, and health status. In addition, those who used homeopathic remedies had a higher proportion of non-intention to vaccinate versus non-users (20.3% vs. 12.3%; *p* < 0.001), with statistically significant differences according to the level of education and health status. Finally, a higher proportion of non-intention to vaccinate was found in those who consumed garlic, ginger, and lemon versus non-users (18.9% vs. 11.9%; *p* < 0.001). In this last group, differences were observed in non-intention to vaccinate according to gender, age, and health status (Table 2).

### 3.7. Association between Using Herbal Supplements to Prevent COVID-19 Infection and Non-Intention from Vaccinating against COVID-19

In the crude model, a higher prevalence of non-intention to get vaccinated against COVID-19 was found in those who took herbal supplements than in those who did not (cPR = 1.49; 95% CI: 1.34–1.65; *p* < 0.001). This association was maintained in the model adjusted for gender, age, educational level, area of residence, and health status (aPR = 1.44; 95% CI: 1.30–1.58; *p* < 0.001) (Table 3).

### 3.8. Association between the Use of Homeopathic Remedies to Prevent COVID-19 Infection and Non-Intention from Vaccinating against COVID-19

In the crude analysis, a higher prevalence of non-intention to get vaccinated against COVID-19 was found in those who used homeopathic remedies than those who did not (cPR = 1.64; 95% CI: 1.31–2.06; *p* < 0.001). This association continued to be statistically significant in the analysis adjusted for gender, age, educational level, area of residence, and health status (aPR = 1.58; 95% CI: 1.25–1.98; *p* < 0.001) (Table 3).

### 3.9. Association between the Consumption of Garlic, Ginger and Lemon to Prevent Infection by COVID-19 and Non-Intention to Vaccinate against COVID-19

The crude analysis found a higher prevalence of non-intention to be vaccinated against COVID-19 in those who consumed garlic, ginger, and lemon than those who did not (cPR = 1.59; 95% CI: 1.56–1.62; *p* < 0.001). This association was maintained after adjusting for gender, age, educational level, area of residence, and health status (aPR = 1.55; 95% CI: 1.50–1.61; *p* < 0.001) (Table 3).

## 4. Discussion

The present study evaluated the association between the consumption of herbal supplements or homeopathic remedies as a means of prevention for COVID-19 with the intention of vaccination for COVID-19 in LAC. The intention to vaccinate against COVID-19 was present in eight out of ten adults in the region. In addition, the use of herbal supplements, homeopathic remedies, and the consumption of garlic, ginger, and lemon to prevent COVID-19 was found to be significantly associated with non-intention to vaccinate against COVID-19.

Regarding CAM use, our study found a lower use rate than that found in other contexts at various times during the pandemic. For example, a survey in Saudi Arabia between May and June 2020 found that the use of herbs and natural products against COVID-19 during the pandemic was 92.7%, with honey, black seeds, lemon, and ginger being the most used [29]. In Hong Kong, 44% of respondents used CAM during the pandemic [29], similar to Ethiopia’s. On the other hand, a survey found that 46.2% of participants used traditional medicines to prevent and treat COVID-19 cases [30]. Another study in India showed that 85.2% used Ayurvedic or homeopathic medicine to maintain health during the pandemic [31].

Moreover, in Hindu patients isolated due to infection, 25.8% used CAM during and after treatment, primarily Ayurvedic medicine and often more than one [32]. The variability in terms of the results could be influenced by methodological and cultural aspects, personal experiences, access to health systems, the impact of COVID 19 in each context, and differences regarding the local use of each product in different countries. Among the methodological aspects, it is worth highlighting the differences in terms of the populations studied since some studies include patients with a history of COVID-19 infection or in quarantine during the first phase of the pandemic, where the probability of worrying about the infection and with it, seek some help, is more significant. Likewise, given the variability found in CAM types, and given that the tool used for our study was not specific, there could be confusion regarding what is considered CAM, as described in the studies evaluating the use of homemade remedies [33,34].

The literature describes different motivations for the use of CAM during the pandemic. For example, in Saudi Arabia, one study reported that 69.3% of the participants stated that they used these products to improve their immunity, but not as protection *per se* or directed against infection, and 8.7% used CAM to alleviate the symptoms of COVID-19, but not to cure the infection [29]. Likewise, 3.8% of the population used CAM to reduce COVID-19 symptoms and cure them, and 1% to protect themselves from infection and did not need to follow any hygiene precautions [29]. In this country, it was described that social networks and the Internet, in general, were the primary motivators for participants to use them, and vitamin C was the most widely used dietary supplement [35].

In Hong Kong, the most widely used products were vitamins or other dietary supplements and Chinese medicinal herbs, generally as a means of “strengthening the immune system” [36]. In a study in England, India, and the United States, it was found that 93% of those interviewed thought that home remedies helped treat COVID-19 or other viral infections and boost immunity [37]. Most took a mixture of Ayurvedic herbs and spices, lemon, or other fruits as a source of vitamin C [37]. Concerning this, although several studies were published on the role of CAM in the management of COVID-19 [38], such as high Andean phytotherapy [39], herbal therapy [40], traditional Chinese medicine [41], and homeopathy [42,43], to date there is no conclusive evidence about its benefit [44,45]. That is due to methodological problems in these studies that even conditioned the retraction of some investigations in international scientific journals [46]. Despite this lack of evidence, the use of CAM can condition a sense of security and increase the probability of rejection of the COVID-19 vaccine as occurs with other vaccines, which would increase exposure to the disease without protection. However, another possibility is that the use of CAM serves as an additional protective measure against COVID-19 rather than an alternative to the vaccine or other available medical care.

It is described that users or doctors who work with CAM have a higher probability of refusing vaccination in general. A study in emergency services in Switzerland found that vaccination refusal was more frequent among CAM users than non-users and among those who consulted physicians practicing herbal medicine, anthroposophic medicine, or homeopathy [47]. Also, in this country, a qualitative study carried out among doctors who used CAM showed that practitioners framed vaccination decisions as choices at individual and family levels rather than focusing on public health benefits and consequences [48]. In Spain, a study showed that hesitancy before vaccination was associated with mistrust in conventional medicine and was higher among CAM users [17].

Likewise, a multinational study of physicians practicing homeopathy, 77% of whom came from Latin American countries, found that 75.6% considered vaccination safe, effective, and necessary, although 12.5% would not recommend it under any circumstance [49]. Some studies have shown similar results specifically for vaccination against COVID-19. For example, a Finnish study showed that people with more distrust of official sources of information and with more support for CAM and non-pharmacological methods were more reluctant to receive the vaccine [50]. In LAC, a study on health personnel from the northeast of Mexico who showed doubts about vaccination found that preference for homeopathy was one of the reasons [18]. What was described would be in line with what was found in our study, indicating that people using CAM have a lower probability of vaccinating against COVID-19, being necessary the development of educational interventions and other strategies to promote vaccination in this population.

Although the World Health Organization does not advise against homeopathy, mentioning this therapy as part of the traditional medicine in some countries [20], states that should not be used to treat several serious diseases [51]. There are statements from official entities such as the Food and Drug Administration of the United States and the National Health Service of the United Kingdom against its use and regulation [52,53]. To date, homeopathy does not have scientific support from good quality controlled clinical trials published in reliable scientific journals, which would allow establishing that its interventions are effective. Furthermore, people who use CAM are possibly part of groups that contribute to misinformation about vaccines [54]. The association between rejection of vaccines and CAM and frequenting commercial websites selling “natural products” suggests that cultural factors may reinforce an anti-vaccination stance by associating vaccines with capitalism, big pharma, and significant company earnings [55]. Indeed, some studies report the high content of misinformation about vaccines on CAM web pages [55].

Given the risk that these practices represent to the control of the pandemic, it is crucial to work on the action frameworks for the use of CAM, including information systems that allow a better understanding of the population’s practices and their consequences on health. It is also urgent to have an effective governance system for the use of CAM. Public health actors must be clear about the limitations of these practices, with messages that must be included in information strategies on COVID. Similarly, it is necessary to prioritize investment in information, communication, and education campaigns to accelerate vaccination in the region. These actions should be the basis of immunization against COVID-19. In addition, they should be integrated into other highly reluctant vaccination schemes, such as those presented for the measles and pneumococcal vaccine, among others [56].

## 5. Limitations

Regarding the study’s limitations, the cross-sectional design does not allow establishing causal relationships among the variables of interest. Likewise, the use of a social network such as Facebook to carry out the survey limits the universe studied to the population with internet access, which would reduce the generalization of results to the population in extreme poverty whose internet access is limited. In turn, the inclusion of variables in the study is limited to those available in the database. Moreover, the data in the survey were obtained by self-reporting, making underreporting of information possible. Despite these limitations, we consider that the analysis of a database of users of a social network widely used in LAC (four out of five inhabitants of this region use Facebook), with data at the country level, is helpful to characterize and study the non-intention to vaccinate against COVID-19 in this region of the world.

## 6. Conclusions

In conclusion, it was found that in the LAC population, the intention to vaccinate against COVID-19 was present in eight out of ten adults. In addition, there is an association between the use of herbal supplements, the use of homeopathic remedies, and the consumption of garlic, ginger, and lemon to prevent COVID-19 infection and not intend to vaccinate against this disease. Given this scenario, the different actors at the governmental, private, and community levels, as well as health professionals, should warn about the use of practices not supported by scientific evidence and promote the development of strategies aimed at promoting vaccination in populations with less intention to receive the vaccine against COVID-19. This is key to increasing efforts to identify groups using CAM to improve communication strategies and increase the intention to vaccinate.

## Figures and Tables

**Table 1:** General characteristics of the study sample: Use of herbal supplements (*n* = 28,590; N = 404,219), use of homeopathic remedies (*n* = 28,566; N = 409,920), the consumption of garlic, ginger, and lemon (*n* = 28,632; N = 402,476).

Characteristics	Use of Herbal Supplements (N = 28,590)			Use of Homeopathic Remedies (N = 28,566)			Eating Garlic, Ginger, or Lemon (N = 28,632)		
	Absolute Frequency	Weighted Proportion *		Absolute Frequency	Weighted Proportion *		Absolute Frequency	Weighted Proportion *	
	*n*	%	95% CI	N	%	95% CI	*n*	%	95% CI
Gender									
Female	15,862	50.0	49.5–50.5	15,864	50.3	49.7–50.8	15,808	49.7	49.3–50.2
Male	12,665	49.8	49.2–50.3	12,622	49.0	47.8–50.2	12,758	50.0	49.5–50.4
Not binary	63	0.2	0.1–0.3	80	0.7	0.3–1.6	66	0.3	0.2–0.4
Age (years)									
18–30	8665	30.8	26.4–35.6	8701	30.3	26.8–34.1	8726	30.2	26.0–34.8
31–40	7035	20.7	19.2–22.4	7064	20.4	20.2–20.7	7051	21.4	20.0–22.9
41–50	5504	18.3	17.6–18.9	5404	17.7	17.6–17.8	5576	18.4	17.6–19.2
51–60	4405	15.7	14.9–16.5	4397	15.9	14.4–17.4	4345	15.6	14.7–16.5
61–70	2285	11.0	9.2–13.1	2357	11.6	9.9–13.4	2300	10.9	9.9–12.1
71–80	621	3.0	2.2–4.2	567	3.3	2.8–4.0	558	3.0	2.1–4.1
80 or more	75	0.5	0.4–0.6	76	0.8	0.4–1.4	76	0.5	0.4–0.6
Education level									
Less than primary school	365	3.7	1.3–10.5	368	3.8	1.2–11.0	362	3.2	1.1–9.1
Primary school	1709	9.8	5.9–15.8	1773	9.6	5.4–16.6	1653	9.1	5.4–15.0
Secondary school	10,829	42.4	36.2–48.8	10,843	42.1	36.5–47.9	10,926	43.3	37.1–49.6
College/University	12,097	33.7	20.8–49.6	12,043	33.6	20.0–50.5	12,115	33.8	20.9–49.7
Graduate school	3590	10.4	8.7–12.4	3539	10.9	8.7–13.5	3576	10.6	8.6–13.0
Living area									
City	23,940	86.2	75.4–92.7	23,791	85.9	74.2–92.8	23,911	86.1	75.5–92.5
Town	3227	9.0	3.4–21.7	3352	9.2	3.3–23.4	3266	9.2	3.4–22.3
Village or rural area	1423	4.8	4.1–5.6	1423	4.9	4.1–5.7	1455	4.8	4.0–5.7
Health condition									
Poor	729	3.2	2.7–3.8	666	3.0	2.1–4.2	713	3.2	2.1–4.8
Fair	4213	18.1	15.9–20.4	4278	17.9	15.8–20.2	4339	17.6	15.5–20.0
Good	9546	34.2	31.9–36.5	9686	34.2	30.4–38.3	9490	33.7	31.6–35.9
Very good	8887	27.0	25.4–28.7	8830	28.0	26.4–29.6	8896	28.0	26.6–29.4
Excellent	5215	17.5	14.1–21.5	5106	16.9	13.5–21.0	5194	17.4	14.0–21.5
Vaccination intention									
Yes	24,602	86.8	83.8–89.3	24,511	87.3	84.0–90.0	24,662	87.3	84.4–89.7
No	3988	13.2	10.7–16.2	4055	12.7	10.0–16.0	3970	12.7	10.3–15.6

95% CI: 95% Confidence Interval. * Weights and the design effect of the complex survey sampling were included.

**Table 2:** General characteristics according to each exposure group to prevent COVID-19 infection in LAC.

Characteristics	Use of Herbal Supplements (N = 28,590)					Use of Homeopathic Remedies (N = 28,566)					Eating Garlic, Ginger, or Lemon (N = 28,632)				
	Yes		No		*p*-Value *	Yes		No		*p*-Value *	Yes		No		*p*-Value *
	n	%	n	%		n	%	n	%		*n*	%	*n*	%	
Vaccination intention															
Yes	1713	81.0	22,889	87.2	<0.001	1290	79.7	23,221	87.7	<0.001	3126	81.1	21,536	88.1	<0.001
No	458	19.0	3530	12.8		340	20.3	3715	12.3		772	18.9	3198	11.9	
Gender															
Female	1238	54.2	14,624	49.7	0.043	885	51.2	14,979	50.2	0.521	2109	53.5	13,699	49.3	<0.001
Male	927	45.4	11,738	50.1		737	48.5	11,885	49.0		1774	46.0	10,984	50.5	
Not binary	6	0.4	57	0.2		8	0.3	72	0.8		15	0.5	51	0.2	
Age (years)															
18–30	584	24.5	8081	31.3	<0.001	516	29.3	8185	30.4	0.052	1085	24.9	7641	31.0	<0.001
31–40	492	19.2	6543	20.9		377	17.6	6687	20.6		894	19.2	6157	21.7	
41–50	456	19.5	5048	18.2		298	19.0	5106	17.7		741	17.3	4835	18.5	
51–60	376	17.0	4029	15.6		278	18.9	4119	15.7		680	18.7	3665	15.2	
61–70	209	15.3	2076	10.7		129	9.9	2228	11.6		388	15.5	1912	10.3	
71–80	52	4.4	569	2.9		28	4.3	539	3.3		97	4.0	461	2.8	
80 or more	2	0.1	73	0.5		4	1.0	72	0.8		13	0.4	63	0.5	
Education level															
Less than primary school	30	3.5	335	3.8	0.022	11	2.6	357	3.9	0.002	48	3.4	314	3.2	0.320
Primary school	110	11.6	1599	9.6		94	8.8	1679	9.6		217	9.6	1436	9.1	
Secondary school	671	35.6	10,158	42.9		503	32.5	10,340	42.6		1378	38.8	9548	43.8	
College/University	1022	36.1	11,075	33.5		768	40.8	11,275	33.2		1746	36.9	10,369	33.4	
Graduate school	338	13.2	3252	10.2		254	15.3	3285	10.7		509	11.3	3067	10.5	
Living area															
City	1752	84.9	22,188	86.3	0.472	1274	82.4	22,517	86.1	0.092	3095	83.6	20,816	86.4	0.297
Town	298	10.5	2929	8.9		237	9.8	3115	9.2		540	11.4	2726	8.9	
Village or rural area	121	4.6	1302	4.8		119	7.8	1304	4.7		263	5.0	1192	4.7	
Health condition															
Poor	72	5.2	657	3.1	0.004	44	3.6	622	3.0	0.013	112	3.5	601	3.2	0.007
Fair	368	19.0	3845	18.0		216	14.8	4062	18.0		643	19.3	3696	17.4	
Good	659	30.9	8887	34.4		492	31.9	9194	34.4		1184	29.1	8306	34.3	
Very good	628	24.1	8259	27.3		514	27.1	8316	28.0		1141	28.3	7755	28.0	
Excellent	444	20.8	4771	17.2		364	22.6	4742	16.6		818	19.7	4376	17.1	

95% CI: 95% Confidence Interval. Weights and the design effect of the complex survey sampling were included. * Refers to the statistical significance obtained from comparing the proportions between the categories of the variables considering the survey’s complex sampling. Bold values denote statistical significance at the *p* < 0.05 level.

**Table 3:** Crude and adjusted regression models to evaluate the association between the use of herbal supplements, the use of homeopathic remedies, the consumption of garlic, ginger, or lemon, and non-intention to vaccinate.

Exposure	No Vaccination Intention			
	Crude Model ^a^		Adjusted Model ^a,b^	
	cPR (95% CI)	*p*-Value	aPR (95% CI)	*p*-Value
Use of herbal supplements				
No	Ref.	---	Ref.	---
Yes	1.49 (1.34–1.65)	<0.001	1.44 (1.30–1.58)	<0.001
Use of homeopathic remedies				
No	Ref.	---	Ref.	---
Yes	1.64 (1.31–2.06)	<0.001	1.58 (1.25–1.98)	0.001
Eating garlic, ginger or lemon				
No	Ref.	---	Ref.	---
Yes	1.59 (1.56–1.62)	<0.001	1.55 (1.50–1.61)	<0.001

cPR: crude prevalence ratio; aPR: adjusted prevalence ratio; 95% CI: 95% Confidence Interval. ^a^ A generalized linear model of the Poisson family was carried out with a link log considering the effect of the design and the weights of the complex sampling of the survey. ^b^ Adjusted for gender, age, education level, living area and health condition.

## Data Availability

Restrictions apply to the availability of these data. Authors obtained the data after signing a contract with the University of Maryland and have not permission to share the database.

## Supplementary Materials

The following are available online at https://www.mdpi.com/article/10.3390/tropicalmed7060095/s1, Table S1: Sensitivity analysis for missing data according to use of herbal supplements in the study sample, Table S2: Sensitivity analysis for missing data according to the use of homeopathic remedies in the study sample, Table S3: Sensitivity analysis for missing data according to eating garlic, ginger or lemon in the study sample, Table S4: Proportion of use of herbal supplements to prevent COVID-19 infection in the Latin America and Caribbean region, Table S5: Proportion of use of homeopathic remedies to prevent COVID-19 infection in the Latin America and Caribbean region, Table S6: Proportion of eating garlic, ginger, and lemon to prevent COVID-19 infection in the Latin America and Caribbean region.
