# Supplementary material for: Consumption of Herbal Supplements or Homeopathic Remedies to Prevent COVID-19 and Intention of Vaccination for COVID-19 in Latin America and the Caribbean
Source: Trop Med Infect Dis. 2022 Jun 8;7(6):95. doi: 10.3390/tropicalmed7060095 (PMC9229216; doi:10.3390/tropicalmed7060095)
Supplement: Supplementary file 1 [file tropicalmed-07-00095-s001.zip › tropicalmed-1738981-supplementary.pdf]

Supplementary material

Table S1. Sensitivity analysis for missing data according to use of herbal supplements in the study sample.

Variables	Use of Herbal Supplements			
	Missings		No missings	
	Weighted proportion		Weighted proportion	
	%	95%CI	%	95%CI
**Vaccination intention**				
Yes	87.7	87.2-88.1	12.3	11.9-12.8
No	87.3	86.8-87.8	12.7	12.2-13.2
**Gender**				
Female	87.7	87.3-88.0	12.3	12.0-12.7
Male	87.6	87.5-87.8	12.4	12.2-12.5
Not binary	90.5	88.6-92.2	9.5	7.8-11.4
**Age (years)**				
18-30	87.8	87.2-88.4	12.2	11.6-12.8
31-40	87.6	87.3-87.8	12.4	12.2-12.7
41-50	87.4	87.1-87.7	12.6	12.3-12.9
51-60	87.7	87.6-87.8	12.3	12.2-12.4
61-70	87.3	86.8-87.8	12.7	12.1-13.2
71-80	88.8	85.8-91.1	11.2	8.9-14.2
80 or more	88.8	86.7-90.6	11.2	9.3-13.3
**Education level**				
Less than primary school	87.9	87.5-88.2	12.1	11.7-12.5
Primary school	87.8	87.3-88.3	12.2	11.7-12.7
Secondary school	87.8	87.5-88.0	12.2	12.0-12.5
College / University	87.5	87.4-87.7	12.5	12.3-12.6
Graduate school	87.2	86.8-87.5	12.8	12.5-13.2
**Living area**				
City	87.6	87.4-87.8	12.4	12.2-12.6
Town	88.0	87.6-88.4	12.0	11.6-12.4
Village or rural area	87.9	87.6-88.2	12.1	11.8-12.4
**Health condition**				
Poor	87.5	86.5-88.5	12.5	11.5-13.5
Fair	87.5	87.2-87.8	12.5	12.2-12.8
Good	87.5	87.2-87.8	12.5	12.2-12.8
Very good	87.8	87.3-88.2	12.2	11.8-12.7
Excellent	87.8	87.5-88.2	12.2	11.8-12.5

95%CI: 95% Confidence Interval.
Weights and the design effect of the complex survey sampling were included.

Table S2. Sensitivity analysis for missing data according to the use of homeopathic remedies in the study sample.

Variables	Use of homeopathic remedies			
	Missings		No missings	
	Weighted proportion		Weighted proportion	
	%	95%CI	%	95%CI
**Vaccination intention**				
Yes	87.4	87.2-87.6	12.6	12.4-12.8
No	87.5	87.3-87.6	12.5	12.4-12.7
**Gender**				
Female	87.6	87.5-87.7	12.4	12.3-12.5
Male	87.4	87.2-87.6	12.6	12.3-12.8
Not binary	77.5	56.0-90.3	22.5	9.7-44.0
**Age (years)**				
18-30	87.7	87.4-88.0	12.3	11.9-12.6
31-40	87.6	86.9-88.2	12.4	11.8-13.1
41-50	87.7	87.5-87.9	12.3	12.1-12.5
51-60	87.1	86.4-87.9	12.9	12.1-13.6
61-70	87.0	86.3-87.5	13.0	12.4-13.7
71-80	87.3	87.0-87.7	12.7	12.3-13.0
80 or more	82.6	76.1-87.7	17.4	12.3-23.9
**Education level**				
Less than primary school	88.1	87.2-88.9	11.9	11.0-12.8
Primary school	87.9	87.6-88.1	12.1	11.8-12.4
Secondary school	87.7	87.5-87.8	12.3	12.2-12.5
College / University	87.4	87.1-87.6	12.6	12.4-12.9
Graduate school	86.4	85.3-87.5	13.6	12.5-14.7
**Living area**				
City	87.4	87.3-87.6	12.6	12.4-12.7
Town	87.5	87.1-88.0	12.5	12.0-12.9
Village or rural area	87.9	86.8-88.8	12.1	11.2-13.2
**Health condition**				
Poor	88.8	88.4-89.1	11.2	10.9-11.6
Fair	87.7	87.3-88.0	12.3	12.0-12.7
Good	87.2	86.8-87.7	12.8	12.3-13.2
Very good	87.1	86.8-87.5	12.9	12.5-13.2
Excellent	88.1	87.7-88.5	11.9	11.5-12.3

95%CI: 95% Confidence Interval.
Weights and the design effect of the complex survey sampling were included.

Table S3. Sensitivity analysis for missing data according to eating garlic, ginger or lemon in the study sample.

Variables	Eating garlic, ginger, or lemon			
	Missings		No missings	
	Weighted proportion		Weighted proportion	
	%	95%CI	%	95%CI
**Vaccination intention**				
Yes	87.7	87.3-88.0	12.3	12.0-12.7
No	87.8	87.2-88.4	12.2	11.6-12.8
**Gender**				
Female	87.7	87.3-88.0	12.3	12.0-12.6
Male	87.6	87.5-87.8	12.4	12.2-12.5
Not binary	91.1	90.0-92.0	8.9	8.0-10.0
**Age (years)**				
18-30	88.1	87.7-88.5	11.9	11.5-12.3
31-40	87.3	87.1-87.5	12.7	12.5-12.9
41-50	87.3	87.2-87.5	12.7	12.5-12.8
51-60	87..5	87.3-87.7	12.5	12.3-12.7
61-70	87.7	87.5-88.0	12.3	12.0-12.5
71-80	88.6	87.6-89.5	11.4	10.5-12.4
80 or more	87.9	84.7-90.5	12.1	9.5-15.3
**Education level**				
Less than primary school	89.1	89.0-89.2	10.9	10.8-11.0
Primary school	88.6	88.3-88.9	11.4	11.1-11.7
Secondary school	87.7	87.4-87.9	12.3	12.0-12.6
College / University	87.4	87.3-87.6	12.6	12.4-12.7
Graduate school	86.9	86.1-87.7	13.1	12.3-13.9
**Living area**				
City	87.6	87.3-87.9	12.4	12.1-12.7
Town	87.8	87.3-88.2	12.2	11.7-12.7
Village or rural area	88.2	87.3-89.0	11.8	11.0-12.7
**Health condition**				
Poor	88.3	87.7-88.8	11.7	11.2-12.3
Fair	87.8	87.4-88.2	12.2	11.8-12.6
Good	87.7	87.5-87.9	12.3	12.1-12.5
Very good	87.3	87.1-87.6	12.7	12.4-12.9
Excellent	87.9	87.2-88.5	12.1	11.5-12.8

95%CI: 95% Confidence Interval.
Weights and the design effect of the complex survey sampling were included.

Table S4. Proportion of use of herbal supplements to prevent COVID-19 infection in the Latin America and Caribbean region.

Countries	Use of Herbal Supplements				
		Yes		No	
		Weighted proportion		Weighted proportion	
	N	%	95%CI	%	95%CI
Argentina	7811	2.6	2.2-3.0	97.4	97.0-97.8
Bolivia	548	19.6	15.7-24.2	80.4	75.8-84.2
Brazil	7503	7.9	7.2-8.8	92.1	91.2-92.8
Chile	560	5.6	3.7-8.2	94.4	91.8-96.2
Colombia	7500	10.0	9.2-11.0	90.0	89.0-90.8
Ecuador	595	15.4	11.9-19.6	84.6	80.3-88.1
Guatemala	610	9.7	7.1-13.2	90.3	86.8-92.9
Honduras	633	15.0	11.6-19.2	85.0	80.8-88.4
Jamaica	445	17.3	13.5-21.8	82.7	78.2-86.5
Mexico	7535	6.5	5.8-7.4	93.5	92.6-94.2
Peru	579	14.1	10.1-19.4	85.9	80.6-90.0
Trinidad & Tobago	585	19.5	16.2-23.3	80.5	76.7-83.8
Uruguay	651	2.0	1.0-3.8	98.0	96.2-99.0
Venezuela	663	18.8	15.4-22.6	81.2	77.4-84.5

95%CI: 95% Confidence Interval.

Weights and the design effect of the complex survey sampling were included.

Table S5. Proportion of use of homeopathic remedies to prevent COVID-19 infection in the Latin America and Caribbean region.

Countries		Use of Homeopathic Remedies			
		Yes		No	
		Weighted proportion		Weighted proportion	
	N	%	95%CI	%	95%CI
Argentina	7768	3.5	3.0-4.1	96.5	95.9-97.0
Bolivia	580	14.6	10.6-19.8	85.4	80.2-89.4
Brazil	7583	4.2	3.7-4.9	95.8	95.1-96.3
Chile	584	4.7	3.1-7.0	95.3	93.0-96,9
Colombia	7718	7.4	6.7-8.2	92.6	91.8-93.3
Ecuador	610	10.9	7.9-15.0	89.1	85.0-92,1
Guatemala	644	9.8	6.4-14.6	90.2	85.4-93.5
Honduras	582	12.1	8.9-16.1	87.9	83.9-91.1
Jamaica	433	12.0	8.9-15.9	88.0	84.1-91.1
Mexico	7447	5.7	5.0-6.4	94.3	93.6-95.0
Peru	585	4.6	2.8-7.6	95.4	92.4-97.2
Trinidad & Tobago	594	21.5	12.5-34.2	78.5	65.8-87.5
Uruguay	624	1.8	1.0-3.3	98.2	96.7-99.0
Venezuela	700	11.4	8.8-14.7	88.6	85.3-91.2

95%CI: 95% Confidence Interval.

Weights and the design effect of the complex survey sampling were included.

Table S6. Proportion of eating garlic, ginger, and lemon to prevent COVID-19 infection in the Latin America and Caribbean region.

Countries		Eating garlic, ginger, or lemon			
		Yes		No	
		Weighted proportion		Weighted proportion	
	N	%	95%CI	%	95%CI
Argentina	7770	7.2	6.5-7.9	92.8	92.1-93.5
Bolivia	559	34.1	29.5-39.1	65.9	60.9-70.5
Brazil	7551	9.0	8.2-9.9	91.0	90.1-91.8
Chile	619	11.0	8.4-14.2	89.0	85.8-91.6
Colombia	7608	21.7	20.5-23.0	78.3	77.0-79.5
Ecuador	570	39.0	33.6-44.7	61.0	55.3-66.3
Guatemala	609	28.8	23.7-34.4	71.2	65.6-76.3
Honduras	605	28.9	24.6-33.7	71.1	66.3-75.4
Jamaica	420	22.5	17.8-28.0	77.5	72.0-82.2
Mexico	7611	15.3	14.1-16.6	84.7	83.4-85.9
Peru	560	28.3	23.6-33.5	71.7	66.5-76.3
Trinidad & Tobago	634	22.8	19.3-26.8	77.2	73.2-80.7
Uruguay	638	9.9	7.3-13.4	90.1	86.6-92.7
Venezuela	662	29.3	25.3-33.7	70.7	66.2-74.7

95%CI: 95% Confidence Interval.

Weights and the design effect of the complex survey sampling were included.
